# Development of actionable quality indicators and an action implementation toolbox for appropriate antibiotic use at intensive care units: A modified-RAND Delphi study

**DOI:** 10.1371/journal.pone.0207991

**Published:** 2018-11-29

**Authors:** Marlot C. Kallen, Marie-Jose Roos-Blom, Dave A. Dongelmans, Jeroen A. Schouten, Wouter T. Gude, Evert de Jonge, Jan M. Prins, Nicolette F. de Keizer

**Affiliations:** 1 Amsterdam UMC, University of Amsterdam, Department of Infectious Diseases, Amsterdam, The Netherlands; 2 Amsterdam UMC, University of Amsterdam, Department of Medical Informatics, Amsterdam Public Health Research Institute, Amsterdam, The Netherlands; 3 National Intensive Care Evaluation (NICE) Foundation, Amsterdam, The Netherlands; 4 Amsterdam UMC, University of Amsterdam, Department of Intensive Care Medicine, Amsterdam, The Netherlands; 5 Canisius Wilhelmina Hospital, Department of Intensive Care Medicine, Nijmegen, The Netherlands; 6 Radboud University Medical Center, Department of Scientific Institute for Quality of Healthcare (IQ healthcare), Nijmegen, The Netherlands; 7 Leiden University Medical Center, Department of Intensive Care Medicine, Leiden, The Netherlands; Institute of Social and Preventive Medicine, SWITZERLAND

## Abstract

**Introduction:**

Extensive antibiotic use makes the intensive care unit (ICU) an important focus for antibiotic stewardship programs. The aim of this study was to develop a set of actionable quality indicators for appropriate antibiotic use at ICUs and an implementation toolbox, which can be used to assess and improve the appropriateness of antibiotic use in the treatment of adult patients at an ICU.

**Methods:**

A four round modified-RAND Delphi procedure was used. Potential indicators were identified by a multidisciplinary panel of 15 Dutch experts, from international literature and guidelines. Using an online survey, the identified indicators were rated on three criteria: relevance, actionability and feasibility. Experts discussed and rated the indicators for the second time during a face-to-face consensus meeting. During a final consensus meeting the toolbox was developed, containing potential barriers and improvement strategies which were identified using a validated checklist by Flottorp et al., and if available also containing supporting material.

**Results:**

The first round resulted in 24 potential indicators. After the final meeting a set of three process indicators, one structure indicator and one quantity metric remained: 1) perform at least two sets of blood cultures before start of empirical systemic therapy; 2) perform therapeutic drug monitoring in patients treated with vancomycin or aminoglycosides; 3) perform surveillance cultures if selective digestive or oropharyngeal decontamination is applied at the ICU; 4) biannual face-to-face meetings between ICU and microbiology staff in which local resistance rates are discussed; and 5) quantitative antibiotic use at the ICU expressed in days of therapy (DOT). The toolbox contains 24 unique barriers and 37 improvement strategies.

**Conclusions:**

Our study identified a set of four actionable quality indicators and one quantity metric, together with an implementation toolbox, to improve appropriate antibiotic use at ICUs.

## Introduction

Infection is a major cause of morbidity and mortality in critically ill patients, resulting in a high percentage of patients using antibiotics in intensive care units (ICUs), up to 71% [1]. Inappropriate use of antibiotics is the main driving force in the emergence and spread of resistant microorganisms, which makes the intensive care an important focus for antibiotic stewardship programs (ASPs) [2]. ASPs are coordinated programs designed to improve the appropriate use of antibiotics at an institutional level [3]. Integration of ASPs in the ICU is essential in order to pursue optimal antibiotic use in critically ill patients.

A requirement for an effective stewardship program is the ability to measure the appropriateness of antibiotic use. Quality indicators are defined as measurable elements designed to evaluate aspects of quality of care [4]. Previous studies have developed indicators to measure appropriate antibiotic use in hospitalized non-ICU adults. These studies showed large variation in quality of antibiotic use between hospitals and thus considerable room for improvement [5–8]. In addition, studies illustrated that the use of quality indicators improves the appropriateness of antibiotic use and is associated with a decreased length of hospital stay [9, 10]. The Organisation for Economic Cooperation and Development (OECD) and the Agency for Healthcare Research and Quality (AHRQ) have defined criteria for good quality indicators: a good indicator should be relevant, actionable, reliable, show room for improvement and data collection should be feasible [11, 12]. Literature points out that actionability, meaning that the indicator offers clear direction to improve performance in daily practice, specifically contributes to the success of quality improvement [13, 14].

Indicator scores can be used to develop tailored interventions. Tailored interventions are designed to achieve improvements in healthcare, based on the assessment of local barriers in clinical practice [15]. Systematic tailoring of improvement strategies entails three key steps: 1) identification of the barriers of practice (e.g. factors that hinder the performance of recommended appropriate antibiotic use), 2) designing interventions targeted at these barriers, and 3) application and assessment of the effects of the interventions [15, 16]. However, several factors such as time and resource constraints, lack of knowledge on how to improve, and insufficient involvement of staff members hamper health care professionals to develop and execute improvement strategies [14, 17]. Current literature suggests that providing a pre-established list with barriers that might hinder performance on a quality indicator and a list with suggested improvement strategies to overcome these barriers can support health care providers during the process of quality improvement [18].

The aim of this study was to develop a set of actionable quality indicators and an implementation toolbox, which can be used to assess and improve the appropriateness of antibiotic use in the treatment of adult patients at an ICU.

## Materials and methods

**Fig 1:**
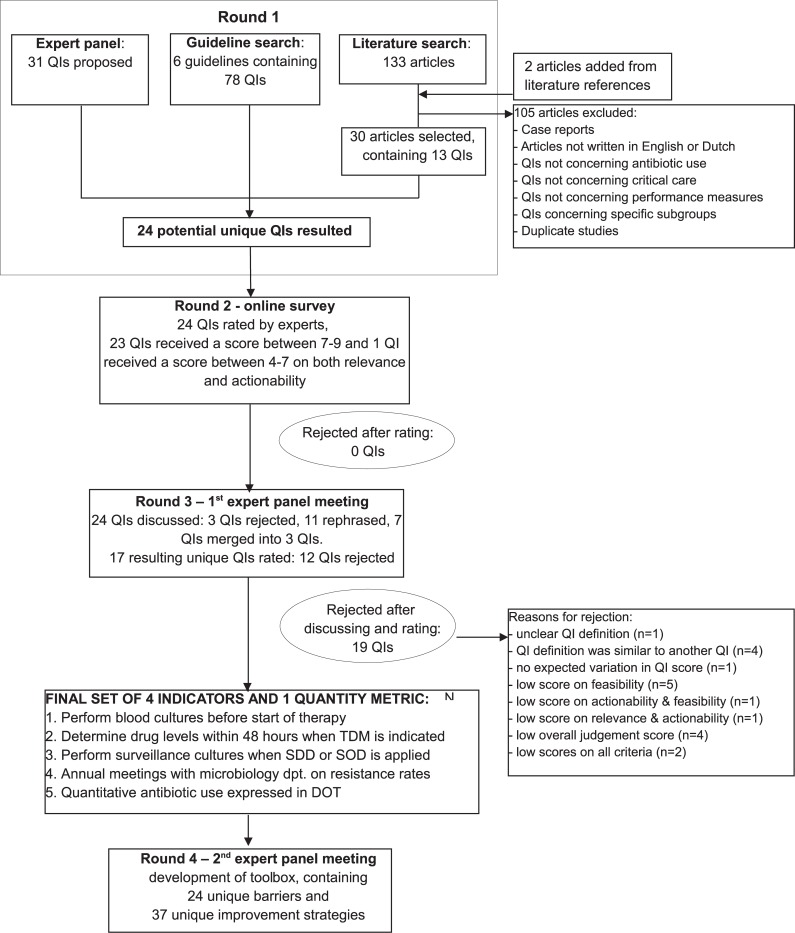
RAND-modified Delphi procedure for the development of quality indicators (QIs) and an action implementation toolbox.

We performed a modified-RAND Delphi procedure, consisting of four rounds, to develop quality indicators and a toolbox for appropriate antibiotic use in the treatment of critically ill patients at an adult ICU [19–21]. An overview of the RAND-modified Delphi procedure is shown in Fig 1.

### Round 1: Identification of potential indicators

First, we performed an inventory of potential indicators among a panel of Dutch experts. The multidisciplinary panel consisted of fifteen experts: three anesthesiologists-intensivists, three internist-intensivists, one intensivist-infectious diseases physician, three internists-ID (infectious diseases) physicians, two clinical microbiologists and three clinical pharmacists. The panelists were invited based on their (inter)national involvement in guideline development, working groups or societies regarding antibiotic use or intensive care (e.g. the National Intensive Care Evaluation (NICE) foundation, the Dutch Working Party on Antibiotic Policy (SWAB) or the European Society of Clinical Microbiology and Infectious Diseases (ESCMID)). They represent nine university and non-university hospitals. The experts were instructed to individually propose potential quality indicators representing appropriate antibiotic use, based on their knowledge and expertise in the field. In addition, we performed a systematic literature search in Medline to identify already available indicators on appropriate antibiotic use. The search included all articles available in Medline up to January 2014. The search was updated in April 2017, which showed no additional indicators relating to antibiotic use at the ICU since 2014. The search strategy is listed in Table 1. Two authors (MK and MJB) independently screened title and abstract in order to identify studies that described (the development of) indicators for appropriate antibiotic use at the ICU. We included all articles that concerned antibiotic use, quality indicators and critical care. Antibiotics were defined as antibacterial agents or antimicrobial agents. Quality indicators were defined as quality measures, performances measures or criteria. Critical care was defined as critical care unit or intensive care unit. Articles were excluded if they were case reports, were not written in English or Dutch, did not concern antibiotic use, critical care, or performance measures, or if they concerned only a specific subgroup of patients. Duplicate studies were removed. We reviewed potentially relevant articles, including references, in full-text format. Third, we made an expert-based selection of national and international guidelines regarding antibiotic use in critically ill patients, from which we extracted all potential indicators (see S1 Appendix). Finally, a list with potential indicators was composed, for which indicators were rephrased if needed and duplicate indicators were removed.

**Table 1:** Search strategy in Medline performed in January 2014. Limits: adults, English, Dutch.

**Quality indicator**	**AND**	**Antibiotic**	**AND**	**Critical care**	**AND**	**Development**
1. quality indicator[Mesh] OR		11. Antibiotic* OR		22. Critical care[Mesh] OR		26. develop*
2. quality criterion OR		12. “Antimicrobial agents” OR		23. Intensive care units[Mesh] OR		
3. quality measure* OR		13. “Antimicrobial drugs” OR		24. “Intensive care medicine” OR		
4. performance indicator OR		14. “Antibacterial drugs” OR		25. Critical illness[Mesh]		
5. performance measure OR		15. “Antibacterial therapy” OR				
6. outcome measure OR		16. “Antimicrobial therapy” OR				
7. outcome indicator* OR		17. Anti-bacterial agents[Mesh] OR				
8. audit OR		18. Anti-infective agents[Mesh] OR				
9. outcome assessment[Mesh] OR		19. “Antibiotic use” OR				
10. process assessment[Mesh]		20. “antimicrobial chemotherapy” OR				
		21. stewardship[tw] OR				
		22. appropriate antibiotic use[tw]				

* truncation symbol = different word variations can be searched for (singular / plural / conjugations).

### Round 2: Online survey

In the second round we converted the list with potential quality indicators into an online survey and instructed experts to appraise each indicator. Based on a 9-point Likert scale (1 = totally disagree, 9 = totally agree), the experts independently rated the potential indicators on two criteria: 1) relevance, the impact of the indicator on disease or on healthcare expenditure and 2) actionability, the extent to which an indicator offers direction for improvement in clinical practice. Indicators with a median score between 4–9 on both relevance and actionability were defined as potentially suitable and indicators with a median score between 1–4 were defined as not suitable [21]. We selected all potentially suitable indicators for the expert panel meeting (see Table 2).

**Table 2:** Results of round 2 and 3 of the RAND-modified Delphi procedure.

Quality indicators	Round 2: Online survey		Round 3: Consensus meeting					Result
	Individual expert rating		Group discussion	Individual expert rating				
	Relevance (median score)	Actionability (median score)		Relevance (median score)	Actionability (median score)	Feasibility (median score	Validity(median score)	
1. quantitative antibiotic use (quantity metric)	7	7	Rephrased	8,5	8	9	8	**Accepted**
2. quantitative use of restricted antibiotics (quantity metric)	7	8	Merged with 1					
3. ratio of restricted versus total antibiotic use	7	7	Merged with 1					
4. costs of antibiotics used	5,5	5	Rejected after discussion					
5. duration of antibiotic therapy	8	8	Rephrased	8	8	2,5	4,5	Rejected
6. adequate duration of antibiotic therapy	8	8	Merged with 5					
7. empirical antibiotic therapy according to the guideline	8	8	Rephrased	8,5	8	6,5	7	Rejected
8. unnecessary vancomycin use	7,5	7,5	Rejected after discussion					
9. obtain blood cultures before start of therapy	8	8,5	Rephrased	9	8,5	7,5	8	**Accepted**
10. obtain site cultures before start of therapy	7,5	8	Rephrased	8,5	7,5	4	5	Rejected
11. adequate drug levels	7	7	Merged with 12					
12. adequate drug level determination for vancomycin	7,5	8	Rephrased	8,5	8,5	9	8	**Accepted**
13. protocol for dose adjustment	8	8		9	9	9	7	Rejected
14. adequate dose adjustment	8	8		9	8	1	4,5	Rejected
15. intravenous-to-oral switch	7	6,5		1	3	7	3	Rejected
16. application of selective digestive or oropharyngeal decontamination	8	8	Rephrased	7,5	7	7,5	6	Rejected
17. obtain blood cultures before application of selective digestive or oropharyngeal decontamination	7	7	Rephrased	9	8	8	8	**Accepted**
18. antimicrobial stewardship specialist present during multidisciplinary meeting	8	8	Rephrased	9	8	8,5	6,5	Rejected
19. protocol for monitoring of resistance	8	8	Rephrased	9	9	8	7,5	**Accepted**
20. local antibiotic guidelines	8	8	Rejected after discussion					
21. pen-to-needle time	8	7,5		9	7	3	5	Rejected
22. turn-around-times of cultures (blood draw to lab result)	8	8		8	2,5	1	3,5	Rejected
23. turn-around-times of cultures (reception of culture at lab to lab result)	8	7		5	1,5	1	2	Rejected
24. documentation of antibiotic plan	7,5	7,5	Rephrased	9	9	7	7	Rejected

### Round 3: Expert panel consensus meeting

During a face-to-face consensus meeting held on October 1^st^ 2015, we presented the results of the second round to the expert panel. All 15 experts from round 2 were invited, and 10 of them were available at the selected meeting date. The indicators were discussed, rephrased, merged and rated for the second time based on three criteria: 1) relevance, 2) actionability and 3) feasibility of data collection, the ability to use routinely collected electronic data. In addition, the experts appraised each indicator with a validity score, reflecting whether the indicator is associated with and appears to measure quality of antibiotic use in clinical practice (i.e. face and content validity). A blinded survey tool was used to support independent rating. After the second rating all indicators with median scores of 7–9 on relevance, actionability and feasibility without disagreement (i.e. 80% of the assessment rates were within the range of the median scores of 7–9), together with a validity score in the highest quartile of all validity scores, were selected (see Table 2) [21]. The selected indicators were described in detail according to the AIRE instrument, including definitions, in-and exclusion criteria, target values, important case-mix variables and subgroups [4]. If an indicator reflected the degree in which antibiotic use was appropriate and was accompanied by a clear target value, it was defined as a quality indicator. If an indicator reflected the volume of antibiotic use, and its outcome only gained value when comparing it among ICUs, it was defined a quantity metric. Results of the consensus meeting were send to all 15 panel members for their final approval.

### Round 4: Action implementation toolbox

Flottorp et al. developed a checklist with all possible barriers for practice and improvement strategies to overcome these barriers, by performing an extensive systematic review of frameworks of determinants of practice followed by a consensus procedure [15]. Based on this validated checklist and expert opinion we defined per selected indicator a list with all possible barriers that can lead to poor performance on that indicator, and a list with improvement strategies to overcome these specific barriers. The list with barriers and improvement strategies was discussed during a second face-to-face meeting. All 15 experts from round 2 were invited, and 5 of them were available at the selected meeting date. The experts adjusted the predefined list and suggested additional barriers and improvement strategies. Again, results of the consensus meeting were send to all 15 panel members for approval and additional suggestions. The final list with barriers and improvement strategies were grouped into four out of the seven predefined categories that are considered relevant in the antibiotic care process [15]. If available, literature or materials, e.g. posters, that could support implementation of the improvement strategies was provided (see S2 Appendix). The barriers, improvement strategies and supporting materials together form “the action implementation toolbox”.

The Medical Ethics Research Committee of the Academic Medical Center confirmed that the Medical Research Involving Human Subjects Acts (WMO) did not apply to this study, since the study subjects were to receive treatment according to standard care and had no burden of the study (August 2016).

## Results

### Round 1: Identification of potential indicators

The expert panel proposed in the first round 31 unique quality indicators for appropriate antibiotic use, based on their clinical experience. The literature search resulted in 133 scientific articles. Based on predefined exclusion criteria 105 articles were excluded. The remaining 28 articles were selected for full-text screening. Two additional articles were selected from literature references and added to the final list. Thirteen indicators were extracted from these studies. Seventy-eight indicators were extracted from six guidelines regarding antibiotic therapy in critically ill patients (S1 Appendix). After de-duplication and rephrasing, a set of 24 potential indicators remained (Fig 1).

### Round 2: Online survey

The panel members rated the 24 potential indicators on their relevance and actionability. All indicators were considered potentially suitable and were therefore selected for the next round (Fig 1 and Table 2).

### Round 3: Expert panel consensus meeting

During a three-hours consensus meeting all 24 indicators were discussed. As a result, three indicators were rejected, eleven indicators were rephrased and seven interrelated indicators were merged into three indicators. One indicator was considered a quantity metric rather than a quality indicator, as this metric provides relevant context to the other indicators despite the absence of a target value. Sixteen resulting unique indicators and the quantity metric were rated for the second time. Thirteen indicators and the quantity metric received a median score of 7 or higher on relevance, actionability and feasibility, of which four indicators and the quantity metric received a validity score in the highest quartile (Fig 1, Table 2).

This resulted in a final set of three process indicators, one structure indicator and one quantity metric: 1) perform at least two sets of blood cultures before start of empirical systemic therapy; 2) perform therapeutic drug monitoring in patients treated with vancomycin or aminoglycosides, within 48 hours after start of antibiotic therapy; 3) perform surveillance cultures if selective digestive or oropharyngeal decontamination is applied at the ICU; 4) biannual face-to -face meetings between IC staff and microbiology staff in which local resistance rates and trends in the ICU population are discussed; and 5) quantitative antibiotic use at the ICU expressed in days of therapy (DOT; one day of therapy represents the administration of a single agent on a given day regardless of the number of doses administered or dosage strength) [22]. Targets for indicator 1, 2 and 3 were set at 100%, which is a theoretical optimum, meaning that it is not necessarily realistic but ICUs should aim to achieve this optimum. The target for indicator 4 was based on expert opinion and set at a minimum of two meetings per year. The quantity metric provides context for the indicators and is suitable for benchmarking ICUs. All 15 panel members approved the detailed description of the selected indicators. The final set of quality indicators and quantity metric is presented in Table 3.

**Table 3:** Final list of actionable quality indicators and quantity metric to monitor appropriate antibiotic use for bacterial infections in hospitalized adult patients admitted at the ICU.

	Quality Indicator	Indicator type	Definition	Numerator	Denominator	Target value
**1**	Perform blood cultures before start of antibiotic therapy	Process	Percentage of patients in whom at least two sets of blood cultures were performed before start of empirical systemic therapy	Number of patients in whom at least 2 sets of blood cultures were performed between 24 hours before and 24 hours after empirical systemic antibiotic therapy was started	Total number of patients who started with empirical systemic antibiotic therapy	100%
**2**	Perform therapeutic drug monitoring in patients treated with vancomycin or aminoglycosides, within 48 hours	Process	Percentage of patients treated with vancomycin or aminoglycosides in whom drug levels were determined within 48 hours after start of antibiotic therapy	Number of patients treated with vancomycin or aminoglycosides in whom drug levels were determined within 48 hours after start of antibiotic therapy	Total number of patients treated with vancomycin or aminoglycosides therapy for at least 48 hours	100%
**3**	Perform surveillance cultures when SDD or SOD is applied	Process	Percentage of patients in whom surveillance cultures were obtained if selective digestive or oropharyngeal decontamination is applied at the ICU	Number of patients in whom at least one surveillance culture was obtained from rectum, throat and airways when selective digestive or oropharyngeal decontamination is applied at the ICU	Total number of patients in whom selective digestive or oropharyngeal decontamination is applied at the ICU	100%
**4**	Biannual face-to -face meetings between IC staff and microbiology staff in which local resistance rates are discussed	Structure	Face-to-face meetings between IC staff and microbiology staff in which local resistance rates and trends in the ICU population are discussed.	-	-	At least 2 times per year
	**Quantity metric**	**Definition**				**Target value**
**5**	Quantitative antibiotic use expressed in DOT	Quantitative antibiotic use at the ICU expressed in days of therapy (DOT) per 100 patient-days or per 100 admissions *Specified for subgroups*: *1) restricted antibiotics*, *2) per diagnosis*				No target value. The metric provides context for indicators and is suitable for benchmarking

### Round 4: Action implementation toolbox

The implementation toolbox shows for each indicator a list of potential barriers in the antibiotic care process, and associated improvement strategies to overcome these barriers. Barriers can be applicable to more than one indicators and improvement strategies can be applicable to more than one barrier. A total of 24 unique barriers and 37 unique improvement strategies were identified and grouped into four categories: A) barriers related to the guidelines, e.g. the local antibiotic guidelines are inadequate or incomplete; B) barriers related to the individual health care professional, e.g. health care professionals are not (sufficiently) familiar with the antibiotic protocol; C) barriers related to professional interactions, e.g. there is insufficient communication within ICU teams; D) barriers related to incentives and resources, e.g. there is a lack of culture media bottles at the ICU ward. Supporting materials were provided for eight of the unique improvement strategies (S2 Appendix).

## Discussion

In this study we used a modified-RAND Delphi procedure to systematically develop a set of four actionable quality indicators and one quantity metric for appropriate antibiotic use in adult ICUs. In addition, we developed an implementation toolbox, containing a list with possible barriers that lead to poor performance on the selected indicators, and a list with improvement strategies to overcome these specific barriers, which can be used to support stewardship actions to increase performance on antibiotic use.

A quantity metric for total antibiotic use was selected next to the four quality indicators, as the experts found it important to include this metric that enables benchmarking and provides context to the other indicators despite the absence of a specific target value.

Previous studies have described several quality indicators for ICUs [23–26]. De Vos et al. developed a general set of indicators for quality of care at the ICU and used a method comparable to ours, including established criteria in their selection process of indicators [24]. Nevertheless, they did not use the criterion actionability, resulting in a set that did not contain clues for quality improvement in daily practice: the set consisted mainly of structure indicators, indicators lacked clear definitions and target values, and little or no variation in outcome of indicators was seen between different hospitals. Furthermore, none of these indicators were related to antibiotic use in the ICU. Berenholtz et al. also developed quality indicators focusing on care provided in the ICU, but they focused on the whole range of treatment specifically in sepsis patients [26]. A recent study by van den Bosch et al. was most similar to our study, as it developed a set of indicators focusing on antibiotic treatment. Their set consists of outcome and process indicators with clear definitions, however, they too did not take into account the actionability of indicators. Moreover, they focused on patients with sepsis on a general medical ward or ICU, while we specifically aimed to develop a set of indicators for the ICU [23]. We have taken into account the limitations of previous studies and focused on the importance of actionability of indicators, in order to develop ICU-specific indicators that give clear direction to improve quality of care [13, 14].

The use of quality indicators to measure the appropriateness of antibiotic use requires extensive data collection from patient records, which is labor intensive [24]. Since the implementation of the NICE registry database [27], the majority of Dutch ICUs upload their patient level data from their local electronic health records (EHRs) or patient data management system (PDMS) through automatic data extractions, which reduces the workload significantly. Our set of indicators had to be suitable for electronic data extraction, which therefore was an important prerequisite to score high on ‘feasibility of data collection’. We realize that this might be different in other countries or settings.

Not every clinical setting needs the same level of improvement. Improvement strategies should therefore be tailored, depending on the local barriers in clinical practice. [15, 28] Literature shows that systematic tailoring of improvement strategies can improve quality of care [29]. To our knowledge we are the first to provide an implementation toolbox that supports ICU health care providers in the process of tailoring improvement strategies, as they can select those potential barriers from the toolbox that are relevant to their own context, after which the toolbox displays the suggested improvement strategies associated with the selected barriers. We aim to continuously expand and improve the toolbox with new or revised barriers, strategies and supporting materials. The validated indicators, together with the quality improvement toolbox, will be implemented in an online quality dashboard as part of the Dutch National Intensive Care Evaluation (NICE) registry [27] and evaluated in future work. The online dashboard will be offered to the participating ICUs, providing for each ICU their performance scores and benchmark information on the antibiotic indicators (e.g. the median score of all participating ICUs and the average score of the top 10% best performing ICUs) accompanied by a list of potential barriers in the antibiotic care process and the associated improvement strategies to improve the performance on these indicators [30].

Our study has several strengths. First, we used a modified-RAND Delphi approach, a systematic rigorous procedure in which scientific evidence and expert opinions are combined. [5, 6, 9, 19, 20]. Our study was consistent with the validated method, nevertheless, we made a valuable modification and extension: in contrary to previous studies, at the start of the procedure we instructed the experts to propose indicators reflecting appropriate antibiotic use without providing them a predefined list of potential indicators from the literature. By this, we ensured that the clinical experts raised those indicators that were most relevant in daily practice, also when these were not (yet) described in the literature. Second, we used a multidisciplinary expert panel, consisting of different specialties relevant for antibiotic use in the ICU. Third, to our knowledge, we are the first to provide an additional implementation toolbox. This tool enables health care professionals to systematically select tailored improvement strategies based on local barriers.

Our study also has some limitations. First, for pragmatic reasons the literature search was performed only in Medline. However, we used a wide range of search terms regarding quality measurements and antibiotics and we therefore assume that the terms should identify those studies reporting on quality indicators. Second, the Medline search was performed in 2014 when the project started. The search was therefore updated on April 5^th^ 2017, which showed no additional indicators relating to antibiotic use at the ICU since 2014. Third, we did not involve ICU nurses or patients in the expert panel. In the Netherlands antibiotic agents are solely prescribed by doctors. We realize that nurses are increasingly involved in antimicrobial stewardship and should be included in future expert panels. Patients were not involved in our expert team as this was logistically challenging, and also given the heterogeneity of reasons for ICU admission. Fourth, while some of our indicators have been described before and are applicable to general wards as well as to the ICU, they were selected because they were considered particularly relevant to the ICU setting based on literature and expert opinion, because they offered direction for improvement in clinical practice and because data collection was considered feasible. Moreover, indicator 3 and 4 are specific for the ICU setting only and have not been described as quality indicators before. Finally, although our indicators have been developed in a national setting, our method of development was according to current international standards and as the indicators were based on international literature, guidelines and consensus of a multidisciplinary team of experts with international experience in their field, we believe that the resulting indicators are generalizable to other ICU settings. Furthermore, one is able to select indicators tailored to one’s own healthcare setting (e.g. some countries do not apply SDD).

The results of our study are a first step towards the use of a new set of actionable quality indicators, together with an action implementation toolbox, to monitor and improve the appropriateness of antibiotic use in adult ICUs. The toolbox will support professionals in selecting improvement strategies that work best in their setting, based on careful assessment of local barriers. Clinimetric properties of the indicators and feasibility in daily practice of electronic data reuse from the EHR or PDMS will be tested during an evaluation study using time series analysis.

## Supporting information

S1 Appendix Literature and guidelines selected in the RAND modified Delphi procedure. (DOCX)

S2 Appendix Quality improvement toolbox containing possible barriers and subsequent improvement actions on each quality indicator. (DOCX)

## Abbreviations

AMR: antimicrobial resistance
AB: antibiotic
QI: quality indicator
ICU: Intensive Care Unit
ASP: Antimicrobial Stewardship Program
DOT: days on therapy
SDD: selective digestive decontamination
SOD: selective oropharyngeal decontamination
HER: electronic health record
PDMS: patient data management system
