# Supplementary material for: Development of actionable quality indicators and an action implementation toolbox for appropriate antibiotic use at intensive care units: A modified-RAND Delphi study
Source: PLoS One. 2018 Nov 29;13(11):e0207991. doi: 10.1371/journal.pone.0207991 (PMC6264509; doi:10.1371/journal.pone.0207991)
Supplement: S1 Appendix — (DOCX) [file pone.0207991.s001.docx]

**S1 Appendix: Literature and guidelines selected in the RAND modified Delphi procedure**

| **Author** | **Publication year** | **Title** |
| --- | --- | --- |
| **Literature** | | |
| Alvarez-Lerma F. et al [1] | 1996 | Modification of empiric antibiotic treatment in patients with pneumonia acquired in the intensive care unit |
| [D'Amico R](https://www.ncbi.nlm.nih.gov/pubmed/?term=D%27Amico%20R%5BAuthor%5D&cauthor=true&cauthor_uid=9554897). et al [2] | 1998 | Effectiveness of antibiotic prophylaxis in critically ill adult patients: systematic review of randomized controlled trials |
| Price J. et al [3] | 1999 | Evaluation of clinical practice guidelines on outcome of infection in patients in the surgical intensive care unit |
| Kollef M. H. et al [4] | 1999 | Inadequate antimicrobial treatment of infections: a risk factor for hospital mortality among critically ill patients |
| Nardi G. et al [5] | 2001 | Reduction in gram-positive pneumonia and antibiotic consumption following the use of a SDD protocol including nasal and oral mupirocin |
| Trouillet J.L. et al [6] | 2002 | Pseudomonas aeruginosa ventilator-associated pneumonia: comparison of episodes due to piperacillin-resistant versus  piperacillin-susceptible organisms |
| Garbino J. et al [7] | 2002 | Prevention of severe candida infections in non-neutropenic, high-risk, critically ill patients: a randomized, double-blind, placebo-controlled  trial in patients treated by selective digestive decontamination |
| Eachempati S.R. et al [8] | 2009 | Does de-escalation of antibiotic therapy for ventilator-associated pneumonia affect the likelihood of recurrent pneumonia or mortality in critically ill surgical patients? |
| Chastre J. et al [9] | 2003 | Comparison of 8 vs 15 days of antibiotic therapy for ventilator-associated pneumonia in adults |
| Houston S. et al [10] | 2003 | Reducing the incidence of nosocomial pneumonia in cardiovascular surgery patients |
| Jacobs S. et al [11] | 2003 | Fluconazole improves survival in septic shock: a randomized double-blind prospective study |
| Hughes M.G. et al [12] | 2004 | Effect of an intensive care unit rotating empiric antibiotic schedule on the development of hospital-acquired infections on the non–intensive care unit ward |
| Hartmann B. et al [13] | 2004 | Review of antibiotic drug use in a surgical ICU: management with a patient data management system for additional outcome analysis in patients staying more than 24 Hours |
| Shaw M.J. [14] | 2005 | Ventilator-associated pneumonia |
| Bianco A. et al [15] | 2005 | Appropriateness of glycopeptide use in a hospital in Italy |
| Siempos I.I. et al [16] | 2007 | Carbapenems for the treatment of immunocompetent adult patients with nosocomial pneumonia |
| Bennett K.M. et al [17] | 2007 | Implementation of antibiotic rotation protocol improves antibiotic susceptibility profile in a surgical intensive care unit |
| Berenholtz S.M. et al [18] | 2007 | Developing quality measures for sepsis care in the ICU |
| Aarts M.W. et al [19] | 2007 | Antibiotic management of suspected nosocomial ICU-acquired infection: does prolonged empiric therapy improve outcome? |
| Roberts J.A. et al [20] | 2008 | Better outcomes through continuous infusion of time-dependent  antibiotics to critically ill patients? |
| Lam S.W. et al [21] | 2009 | Evolving role of early antifungals in the adult intensive care unit |
| Garcin F. et al [22] | 2010 | Non-adherence to guidelines: an avoidable cause of failure of empirical antimicrobial therapy in the presence of difficult-to-treat  bacteria |
| Zilberberg M.D. et al [23] | 2010 | Inappropriate empiric antifungal therapy for candidemia in the ICU and hospital resource utilization: a retrospective cohort study |
| Rajamani A. et al [24] | 2011 | The SCRIPT project: a knowledge translation approach to improve prescription practice in a general intensive care unit |
| Mangino J.E. et al [25] | 2011 | Development and implementation of a performance improvement project in adult intensive care units: overview of the Improving  Medicine Through Pathway Assessment of Critical Therapy in Hospital-Acquired Pneumonia (IMPACT-HAP) study |
| Zahar J. et al [26] | 2011 | Outcomes in severe sepsis and patients with septic shock: Pathogen species and infection sites are not associated with mortality |
| Joung M. et al [27] | 2011 | Impact of de-escalation therapy on clinical outcomes for intensive care unit-acquired pneumonia |
| Chang H.J. et al [28] | 2011 | Risk factors and outcomes of carbapenem nonsusceptible Escherichia coli bacteremia: A matched case control study |
| van den Bosch C.M.A et al [29] | 2014 | Development of quality indicators for antimicrobial treatment in adults with sepsis |
| Leone M. et al [30] | 2014 | De-escalation versus continuation of empirical antimicrobial treatment in severe sepsis: a multicenter non-blinded randomized  noninferiority trial |
| **Guidelines** | | |
| Dellit T.H. et al [31] | 2007 | Infectious Diseases Society of America and the Society for Healthcare Epidemiology of America Guidelines for developing an institutional program to enhance Antimicrobial Stewardship |
| Dutch Working Party on Antibiotic Policy [32] | 2010 | SWAB guidelines for antibacterial therapy of adult patients with sepsis |
| Dellinger R.P. et al [33] | 2013 | Surviving Sepsis Campaign: international guidelines for management of severe sepsis and septic shock |
| Oostdijk E.A.N. [34] | 2014 | SWAB guidelines for selective decontamination in patients admitted to the intensive care |
| National Institute for Health and Care Excellence [35] | 2015 | Antimicrobial Stewardship: systems and processes for effective antimicrobial medicine use |
| Schuts E.C. et al [36] | 2015 | Current evidence on hospital antimicrobial stewardship objectives: a systematic review and meta-analysis |

**REFERENCES**

[1] Alvarez-Lerma F. Modification of empiric antibiotic treatment in patients with pneumonia acquired in the intensive care unit. ICU-Acquired Pneumonia Study Group. Intensive care medicine 1996;22(5):387-94.

[2] D'Amico R, Pifferi S, Leonetti C, Torri V, Tinazzi A, Liberati A. Effectiveness of antibiotic prophylaxis in critically ill adult patients: systematic review of randomised controlled trials. BMJ (Clinical research ed) 1998;316(7140):1275-85.

[3] Price J, Ekleberry A, Grover A, Melendy S, Baddam K, McMahon J, et al. Evaluation of clinical practice guidelines on outcome of infection in patients in the surgical intensive care unit. Critical care medicine 1999;27(10):2118-24.

[4] Kollef MH, Sherman G, Ward S, Fraser VJ. Inadequate antimicrobial treatment of infections: a risk factor for hospital mortality among critically ill patients. Chest 1999;115(2):462-74.

[5] Nardi G, Di Silvestre AD, De Monte A, Massarutti D, Proietti A, Grazia Troncon M, et al. Reduction in gram-positive pneumonia and antibiotic consumption following the use of a SDD protocol including nasal and oral mupirocin. European journal of emergency medicine : official journal of the European Society for Emergency Medicine 2001;8(3):203-14.

[6] Trouillet JL, Vuagnat A, Combes A, Kassis N, Chastre J, Gibert C. Pseudomonas aeruginosa ventilator-associated pneumonia: comparison of episodes due to piperacillin-resistant versus piperacillin-susceptible organisms. Clinical infectious diseases : an official publication of the Infectious Diseases Society of America 2002;34(8):1047-54.

[7] Garbino J, Lew DP, Romand JA, Hugonnet S, Auckenthaler R, Pittet D. Prevention of severe Candida infections in nonneutropenic, high-risk, critically ill patients: a randomized, double-blind, placebo-controlled trial in patients treated by selective digestive decontamination. Intensive care medicine 2002;28(12):1708-17.

[8] Eachempati SR, Hydo LJ, Shou J, Barie PS. Does de-escalation of antibiotic therapy for ventilator-associated pneumonia affect the likelihood of recurrent pneumonia or mortality in critically ill surgical patients? The Journal of trauma 2009;66(5):1343-8.

[9] Chastre J, Wolff M, Fagon JY, Chevret S, Thomas F, Wermert D, et al. Comparison of 8 vs 15 days of antibiotic therapy for ventilator-associated pneumonia in adults: a randomized trial. Jama 2003;290(19):2588-98.

[10] Houston S, Gentry LO, Pruitt V, Dao T, Zabaneh F, Sabo J. Reducing the incidence of nosocomial pneumonia in cardiovascular surgery patients. Quality management in health care 2003;12(1):28-41.

[11] Jacobs S, Price Evans DA, Tariq M, Al Omar NF. Fluconazole improves survival in septic shock: a randomized double-blind prospective study. Critical care medicine 2003;31(7):1938-46.

[12] Hughes MG, Evans HL, Chong TW, Smith RL, Raymond DP, Pelletier SJ, et al. Effect of an intensive care unit rotating empiric antibiotic schedule on the development of hospital-acquired infections on the non-intensive care unit ward. Critical care medicine 2004;32(1):53-60.

[13] Hartmann B, Junger A, Brammen D, Rohrig R, Klasen J, Quinzio L, et al. Review of antibiotic drug use in a surgical ICU: management with a patient data management system for additional outcome analysis in patients staying more than 24 hours. Clinical therapeutics 2004;26(6):915-24; discussion 04.

[14] Shaw MJ. Ventilator-associated pneumonia. Current opinion in pulmonary medicine 2005;11(3):236-41.

[15] Bianco A, Rizza P, Scaramuzza G, Pavia M. Appropriateness of glycopeptide use in a hospital in Italy. International journal of antimicrobial agents 2006;27(2):113-9.

[16] Siempos, II, Vardakas KZ, Manta KG, Falagas ME. Carbapenems for the treatment of immunocompetent adult patients with nosocomial pneumonia. The European respiratory journal 2007;29(3):548-60.

[17] Bennett KM, Scarborough JE, Sharpe M, Dodds-Ashley E, Kaye KS, Hayward TZ, 3rd, et al. Implementation of antibiotic rotation protocol improves antibiotic susceptibility profile in a surgical intensive care unit. The Journal of trauma 2007;63(2):307-11.

[18] Berenholtz SM, Pronovost PJ, Ngo K, Barie PS, Hitt J, Kuti JL, et al. Developing quality measures for sepsis care in the ICU. Joint Commission journal on quality and patient safety 2007;33(9):559-68.

[19] Aarts MA, Brun-Buisson C, Cook DJ, Kumar A, Opal S, Rocker G, et al. Antibiotic management of suspected nosocomial ICU-acquired infection: does prolonged empiric therapy improve outcome? Intensive care medicine 2007;33(8):1369-78.

[20] Roberts JA, Lipman J, Blot S, Rello J. Better outcomes through continuous infusion of time-dependent antibiotics to critically ill patients? Current opinion in critical care 2008;14(4):390-6.

[21] Lam SW, Eschenauer GA, Carver PL. Evolving role of early antifungals in the adult intensive care unit. Critical care medicine 2009;37(5):1580-93.

[22] Garcin F, Leone M, Antonini F, Charvet A, Albanese J, Martin C. Non-adherence to guidelines: an avoidable cause of failure of empirical antimicrobial therapy in the presence of difficult-to-treat bacteria. Intensive care medicine 2010;36(1):75-82.

[23] Zilberberg MD, Kollef MH, Arnold H, Labelle A, Micek ST, Kothari S, et al. Inappropriate empiric antifungal therapy for candidemia in the ICU and hospital resource utilization: a retrospective cohort study. BMC infectious diseases 2010;10:150.

[24] Rajamani A, Suen S, Phillips D, Thomson M. The SCRIPT project: a knowledge translation approach to improve prescription practice in a general intensive care unit. Critical care and resuscitation : journal of the Australasian Academy of Critical Care Medicine 2011;13(4):245-51.

[25] Mangino JE, Peyrani P, Ford KD, Kett DH, Zervos MJ, Welch VL, et al. Development and implementation of a performance improvement project in adult intensive care units: overview of the Improving Medicine Through Pathway Assessment of Critical Therapy in Hospital-Acquired Pneumonia (IMPACT-HAP) study. Critical care (London, England) 2011;15(1):R38.

[26] Zahar JR, Timsit JF, Garrouste-Orgeas M, Francais A, Vesin A, Descorps-Declere A, et al. Outcomes in severe sepsis and patients with septic shock: pathogen species and infection sites are not associated with mortality. Critical care medicine 2011;39(8):1886-95.

[27] Joung MK, Lee JA, Moon SY, Cheong HS, Joo EJ, Ha YE, et al. Impact of de-escalation therapy on clinical outcomes for intensive care unit-acquired pneumonia. Critical care (London, England) 2011;15(2):R79.

[28] Chang HJ, Hsu PC, Yang CC, Kuo AJ, Chia JH, Wu TL, et al. Risk factors and outcomes of carbapenem-nonsusceptible Escherichia coli bacteremia: a matched case-control study. Journal of microbiology, immunology, and infection = Wei mian yu gan ran za zhi 2011;44(2):125-30.

[29] van den Bosch CM, Hulscher ME, Natsch S, et al. Development of quality indicators for antimicrobial treatment in adults with sepsis. BMC infectious diseases 2014;14:345.

[30] Leone M, Bechis C, Baumstarck K, Lefrant JY, Albanese J, Jaber S, et al. De-escalation versus continuation of empirical antimicrobial treatment in severe sepsis: a multicenter non-blinded randomized noninferiority trial. Intensive care medicine 2014;40(10):1399-408.

[31] Dellit TH, Owens RC, McGowan JE, et al. Infectious Diseases Society of America and the Society for Healthcare Epidemiology of America guidelines for developing an institutional program to enhance antimicrobial stewardship. Clinical infectious diseases : an official publication of the Infectious Diseases Society of America 2007;44:159-77.

[32] Dutch Working Party on Antibiotic Policy (SWAB). Guidelines for Antibacterial therapy of adult patients with Sepsis: <http://www.swab.nl/swab/cms3.nsf/uploads/65FB380648516FF2C125780F002C39E2/$FILE/swab_sepsis_guideline_december_2010.pdf>. 2010.

[33] Dellinger RP, Levy MM, Rhodes A, Annane D, Gerlach H, Opal SM, et al. Surviving sepsis campaign: international guidelines for management of severe sepsis and septic shock: 2012. Critical care medicine 2013;41(2):580-637.

[34] Oostdijk EAN. Selective decontamination in ICU patients: Dutch guideline. 2015.

[35] National Institution for Health and Care Excellence (NICE). Antimicrobial Stewardship: systems and processes for effective antimicrobial medicine use; <https://www.nice.org.uk/guidance/ng15>. 2015.

[36] Schuts EC, Hulscher ME, Mouton JW, Verduin CM, Stuart JW, Overdiek HW, et al. Current evidence on hospital antimicrobial stewardship objectives: a systematic review and meta-analysis. The Lancet Infectious diseases 2016.
