# Supplementary material for: Development of actionable quality indicators and an action implementation toolbox for appropriate antibiotic use at intensive care units: A modified-RAND Delphi study
Source: PLoS One. 2018 Nov 29;13(11):e0207991. doi: 10.1371/journal.pone.0207991 (PMC6264509; doi:10.1371/journal.pone.0207991)
Supplement: S2 Appendix — (DOCX) [file pone.0207991.s002.docx]

**S2 Appendix: Quality improvement toolbox containing possible barriers and subsequent improvement actions on each quality indicator**

**Local guidelines** = facility-specific evidence based treatment recommendations that assists professionals in their decision making on diagnostics and treatment for a specific disease

1. **Barriers relating to the guidelines**

| **Barrier 1: Antibiotic guidelines are missing** | | |
| --- | --- | --- |
| **Applicable to indicator: 1 - 2 - 3 - 4** | | |
| **Improvement strategy** | **Rationale or description** | **Supporting material** |
| 1. Develop local antibiotic guidelines* specified for the ICU | Empirical therapy according to the guidelines reduces mortality, duration of therapy and length of hospital stay. [1] Locally developed guidelines, based on (inter) national protocols, often have the best chance of being accepted by local health care providers and hence of being implemented [2, 3].  **Local antibiotic guidelines: facility-specific evidence- based treatment recommendations that assist professionals in their decision making* | The Dutch Antibiotic Working Party (SWAB) offers national antibiotic guidelines, which can be adapted for each hospital based on local resistance patterns. http://[www.swabid.nl](http://www.swabid.nl).  SWAB offers Dutch protocols for sepsis and selective digestive or oropharyngeal decontamination. http://www.swab.nl/richtlijnen  The surviving sepsis campaign protocols offer recommendations for the care of severely septic patients [4].  The Infectious Diseases Society of America published many protocols, including the protocol for developing an institutional program to enhance Antimicrobial Stewardship [5]. |

| **Barrier 2: Antibiotic guidelines are inadequate or unclear** | | |
| --- | --- | --- |
| **Applicable to indicator: 1 - 2 - 3 - 4** | | |
| **Improvement strategy** | **Rationale or description** | **Supporting material** |
| 1. Revise antibiotic guidelines so that the recommendations are adequate and understandable | A Dutch study illustrated that adjusting local guidelines based on scientific evidence and consistency with other guidelines, ensuring a better distribution and education on these guidelines, and facilitating the logistics achieved a significant improvement in the quality of care [6]. |  |
| 1. Extend antibiotic guidelines by adding missing criteria or recommendations | **Indicator 1**: Blood cultures should be taken before the start of therapy, since the use of antibiotics lowers the positivity rates [7, 8].  **Indicator 2**: Critically ill patients may have pathology that results in altered pharmacokinetics and renal clearance. Therapeutic drug monitoring (TDM) serves as an accurate method for dose adjustment in critically ill patients. The clinical outcome benefits of a systematic TDM program for antibiotics have mainly been demonstrated for aminoglycosides [9, 10].  **Indicator 3:** Surveillance cultures are needed to determine whether SDD / SOD is effective. If not effective, the frequency of SDD/SOD should be increased or passage through the gastrointestinal tract should be improved.  Controversy exists whether SDD and SOD increase the prevalence of antibiotic resistant bacteria. Therefore, it is advised to accompany the implementation of SDD and SOD with a stringent surveillance system to detect antibiotic resistant pathogens in the ICU. [11]  **Indicator 4:** Up to 71% of patients at an ICU use antibiotics. [12] Extensive use of antibiotics is the main driving force in the emergence of resistant microorganisms. [13] Awareness of (trends) in resistance rates can lead to early detection and initiation of prevention policies. | The Dutch Antibiotic Working Party (SWAB) offers national antibiotic guidelines, which can be adapted for each hospital based on local resistance patterns. http://[www.swabid.nl](http://www.swabid.nl).  The website <http://www.A-teams.nl> offers a list with restricted antibiotics.  SWAB offers Dutch protocols for selective digestive or oropharyngeal decontamination, among others <http://www.swab.nl/richtlijnen>  The Dutch Association for Hospital Pharmacists (NVZA) offers guidelines for TDM: http://tdm-monografie.org/ |

| **Barrier 3: Local antibiotic guidelines are not consistent with the national guidelines** | | |
| --- | --- | --- |
| **Applicable to indicator: 1 - 2 - 3 - 4** | | |
| **Improvement strategy** | **Rationale or description** | **Supporting material** |
| 1. Revise antibiotic guidelines so that the recommendations are consistent with the national guidelines | A Dutch study illustrated that adjusting the local guidelines based on scientific evidence and consistency with other guidelines, ensuring a better distribution and education on these guidelines, and facilitating the logistics achieved a significant improvement in the quality of care [6]. | The Dutch Antibiotic Working Party (SWAB) offers national guidelines, which can be adapted for each hospital based on local resistance patterns. http://[www.swabid.nl](http://www.swabid.nl).  SWAB offers Dutch guidelines for selective digestive or oropharyngeal decontamination, among others <http://www.swab.nl/richtlijnen> |
| 1. Discuss the reasons for conflicting recommendations with the healthcare professionals | Interactive (thematic) meetings can result in more support [14, 15]. |  |

| **Barrier 4: The quality of evidence or source supporting antibiotic guidelines is unclear or weak** | | |
| --- | --- | --- |
| **Applicable to indicator: 1 - 2 - 3 - 4** | | |
| **Improvement strategy** | **Rationale or description** | **Supporting material** |
| 1. Evaluate and discuss recommendations in antibiotic guidelines for which there is low quality evidence | Interactive (thematic) meetings can result in more support [14, 15]. |  |
| 1. Ensure that antibiotic guidelines are made or endorsed by an organisation and people that have credibility with the targeted healthcare professionals | The Dutch Working Party on Antibiotic Policy (SWAB) consists of a multidisciplinary group of experts, consisting of infectious diseases specialists, medical microbiologists, hospital pharmacists, and, if indicated, other specialties. SWAB develops and updates national antibiotic guidelines. | The Dutch Antibiotic Working Party (SWAB) offers national antibiotic guidelines, which can be adapted for each hospital based on local resistance patterns. http://[www.swabid.nl](http://www.swabid.nl). |

| **Barrier 5: The antibiotic guidelines are not (easily) accessible** | | |
| --- | --- | --- |
| **Applicable to indicator: 1 - 2 - 3 - 4** | | |
| **Improvement strategy** | **Rationale or description** | **Supporting material** |
| 1. Ensure that antibiotic guidelines are electronically accessible | Antibiotic guidelines can be easily accessed when available electronically. For example, on a network or online, preferably linked to the electronic health record (EHR) or patient data management system (PDMS) [16].  Use of a computerized system to guide antibiotic administration had shown to minimize adverse drug effects and to reduce inadequate administration of antibiotics [3]. |  |
| 1. Develop a flowchart or pocket card with the highlights of the antibiotic guidelines | Antibiotic guidelines can be easily applied, also in hospitals without EHRs, by offering a flowchart or pocket card covering essential information from the guidelines [16]. | Standard format flowchart or pocket card |
| 1. Spread a digital newsletter with details or updates of the antibiotic guidelines | Information spread by email is an effective way to introduce people to (updates of) the antibiotic guidelines [17]. | Standard format digital newsletter |
| 1. Distribute promotional posters on specific topics relating to antibiotic use | Promotional posters can stimulate familiarity with guidelines and serve as a reminder [18]. | Standard format posters |

| **Barrier 6: The recommendation to discontinue/switch/streamline antibiotics is not feasible for the healthcare professional** | | |
| --- | --- | --- |
| **Applicable to indicator: 1 - 2 - 3 - 4** | | |
| **Improvement strategy** | **Rationale or description** | **Supporting material** |
| 12. Ensure that the clinical action fits into daily practice | Clinical actions that fit into daily practice will be applied more easily. |  |
| 13 + 14 + 15 |  |  |

| **Barrier 7: The recommendation to perform cultures (in time) is not feasible for the healthcare professional** | | |
| --- | --- | --- |
| **Applicable to indicator: 1 - 2 - 3 - 4** | | |
| **Improvement strategy** | **Rationale or description** | **Supporting material** |
| 12 + 13 + 14 + 15 |  |  |

| **Barrier 8: The recommendation to plan annual meetings on antibiotic resistance trends is not feasible for the healthcare professional** | | |
| --- | --- | --- |
| **Applicable to indicator: 1 - 2 - 3 - 4** | | |
| **Improvement strategy** | **Rationale or description** | **Supporting material** |
| 12 + 13 + 14 + 15 |  |  |

**B. Barriers relating to the individual health care professional**

| **Barrier 9: Health care professionals are not (sufficiently) familiar with antibiotic guidelines** | | |
| --- | --- | --- |
| **Applicable to indicator: 1 - 2 - 3 - 4** | | |
| **Improvement strategy** | **Rationale or description** | **Supporting material** |
| 1. Academic detailing: organize a tailored educational meeting on the contents and importance of appropriate antibiotic use | Education will help to raise awareness of antibiotic guidelines, and encourage discussion on the importance of diagnostics and therapy of infectious diseases [19].  (Continuing) educational meetings or discussion groups for all professionals (specialists, residents, nurses) might help to optimize antibiotic prescribing [20].  It might be helpful to point out a coordinator responsible for the educational sessions [17, 21]. | Standard format presentations on topics relating to appropriate antibiotic use at the ICU |
| 1. Organize an interactive meeting or consensus process on specific topics relating to appropriate antibiotic use | Interactive (thematic) meetings can result in more support [14, 15].  It might be helpful to point out a coordinator responsible for the educational sessions [17, 21]. |  |
| 1. Distribute educational material on appropriate antibiotic use | A review on six interventions which evaluated the dissemination of educational materials in printed form or via educational meetings showed positive results on antibiotic prescribing and costs [20]. |  |
| 8 + 9 + 10 + 11 |  |  |

| **Barrier 10: Health care professionals perceive guidelines in general as restricting their autonomy***  *The targeted healthcare professionals may interpret the quality of the evidence or its applicability differently, may not think the recommended intervention is cost-effective, or may lack confidence in the developer of guidelines* | | |
| --- | --- | --- |
| **Applicable to indicator: 1 - 2 - 3 - 4** | | |
| **Improvement strategy** | **Rationale or description** | **Supporting material** |
| 13 + 14 + 15 |  |  |

| **Barrier 11: Health care professionals lack knowledge on the importance of using antibiotics appropriately** | | |
| --- | --- | --- |
| **Applicable to indicator: 1 - 2 - 3 - 4** | | |
| **Improvement strategy** | **Rationale or description** | **Supporting material** |
| 1. Engage leaders or managers in designing and implementing the interventions regarding appropriate antibiotic use | Opinions and behavior of others might reinforce desired practice [22]. |  |
| 1. Appoint a ‘role model’ for appropriate antibiotic use | A role model or opinion leader is able to point out the importance and responsibility for appropriate antibiotic use. Alone or in combination with other interventions they may successfully promote evidence-based practice [21]. |  |
| 1. Ensure there is structural audit and feedback on individual performance | If staff is fed back on their performance on antibiotic use, they will be more aware of the problem [17]. Interventions that included feedback were more effective than those that did not [23]. |  |
| 13 + 14 + 15 |  |  |

| **Barrier 12: Appropriate antibiotic use is not considered important/relevant on the ICU** | | |
| --- | --- | --- |
| **Applicable to indicator: 1 - 2 - 3 - 4** | | |
| **Improvement strategy** | **Rationale or description** | **Supporting material** |
| 13 + 14 + 15 + 16 + 17 + 18 |  |  |

| **Barrier 13: Health care professionals do not agree with the antibiotic guidelines** | | |
| --- | --- | --- |
| **Applicable to indicator: 1 - 2 - 3 - 4** | | |
| **Improvement strategy** | **Rationale or description** | **Supporting material** |
| 13 + 14 + 15 |  |  |

| **Barrier 14: Health care professionals do not believe that appropriate antibiotic use will lead to desired/better outcomes (lack of motivation)** | | |
| --- | --- | --- |
| **Applicable to indicator: 1 - 2 - 3 - 4** | | |
| **Improvement strategy** | **Rationale or description** | **Supporting material** |
| 13 + 14 + 15 + 18 |  |  |

| **Barrier 15: Health care professionals forget to (routinely) perform specific actions regarding appropriate antibiotic use** | | |
| --- | --- | --- |
| **Applicable to indicator: 1 - 2 - 3 - 4** | | |
| **Improvement strategy** | **Rationale or description** | **Supporting material** |
| 1. Install a clinical decision support system in the EHR or PDMS | Information from computer-based medical records can be used to help improve physicians' selection of empiric antibiotics or diagnostics [3, 23, 24]. |  |
| 1. Build in a notification or reminder system in the EHR or PDMS | A review on interventions using reminders showed a reduction in antibiotic prescribing and improvement of appropriate antibiotic use [20]. |  |
| 1. Build in a pre-or post-authorization system for restricted antibiotics in the EHR or PDMS | An infectious disease or microbiology specialist has to approve on a restricted antibiotic, notification of the reason for choosing the restricted antibiotic is mandatory [25]. |  |
| 1. Implement a checklist for actions to be taken along the antibiotic pathway | Use of an antibiotic checklist resulted in a significant increase in appropriateness of antibiotic use [26, 27]. |  |
| 1. Give nurses more responsibility to perform actions regarding antibiotic therapy | If nurses have more independence or responsibility, cultures and TDM can be performed faster and more efficiently, because there is no need to wait for the doctor. The responsibilities of nurses can be detailed in antibiotic guidelines [28]. |  |
| 13 + 14 + 15 + 18 |  |  |

| **Barrier 16: Health care professionals lack skills to perform the actions regarding appropriate antibiotic use** | | |
| --- | --- | --- |
| **Applicable to indicator: 1 - 2 - 3** | |  |
| **Improvement strategy** | **Rationale or description** | **Supporting material** |
| 1. Organize training sessions on skills needed to perform blood cultures or TDM adequately | Organize combined training sessions for specialists, residents, nurses, management coordinators. When training / consultation sessions are organized where the instruments are explained and discussed in difficult situations, the threshold to use the tools can be reduced or eliminated [19].  It might help to point out a coordinator responsible for the educational sessions [17, 21]. |  |
| 13 + 14 + 15 + 18 |  |  |

**C. Barriers relating to professional interactions**

| **Barrier 17: There is inadequate communication or interaction within ICU teams** | | |
| --- | --- | --- |
| **Applicable to indicator: 1 - 2 - 3** | | |
| **Improvement strategy** | **Rationale or description** | **Supporting material** |
| 1. Organize an educative team building event | Educational sessions will help to raise awareness of antibiotic policies, and encourage discussion on the importance of actions regarding the diagnostic process and treatment of infectious diseases [19].  It might help to point out a coordinator responsible for the educational sessions [17, 21]. |  |
| 1. Organize standard evaluation moments with the medical staff | If a team is fed back on their communication and actions regarding the diagnostic process and treatment of antibiotics, they will be more aware of the problem [17]. |  |
| 1. Organize professional teams in a way that roles are defined and members have a shared goal | Substitution of tasks and implementing a shared goal can be effective in attaining better communication [29]. |  |
| 1. Ensure that antibiotic duration is communicated during daily rounds, multidisciplinary meetings, and shift change | A clear and sufficiently communicated treatment plan can lead to better patient outcomes such as a shorter treatment duration [29]. |  |
| 13 + 14 + 15 + 17 + 18 + 22 + 23 + 30 + 31 |  |  |

| **Barrier 18: There is no standard evaluation moment of ICU teams with the Microbiology department about the resistance rates** | | |
| --- | --- | --- |
| **Applicable to indicator: 4** | | |
| **Improvement strategy** | **Rationale or description** | **Supporting material** |
| 1. Schedule evaluation moments | An comfortable team climate is a determinant for success [30].  Discussing local epidemiology can guide the selection of empiric therapy [31]. |  |

| **Barrier 19: The action regarding antibiotic use is not documented in the patient record** | | |
| --- | --- | --- |
| **Applicable to indicator: 1 - 2 - 3 - 4** | | |
| **Improvement strategy** | **Rationale or description** | **Supporting material** |
| 1. Ensure that all performed actions are recorded in the patient record | Ensure that actions regarding antibiotic therapy are always recorded. This gives insight and ensures better communication [19]. |  |
| 1. Build in a (mandatory) documentation area in the EHR or PDMS | A (mandatory) registration area ensures better communication, especially when different health care professionals work in shifts [17]. |  |

**D. Barriers relating to incentives and resources**

| **Barrier 20: Resources for blood or site culture performance are not available** | | |
| --- | --- | --- |
| **Applicable to indicator: 1 - 3** | | |
| **Improvement strategy** | **Rationale or description** | **Supporting material** |
| 1. Ensure that enough culture media are available on the ICU | The availability of culture media can contribute to higher performance of blood or site cultures, which can improve the appropriateness of antibiotic therapy. |  |
| 1. Ensure that the culture media can be found easily | Easy access to culture media can contribute to higher performance of blood or site cultures, which can improve the appropriateness of antibiotic therapy. |  |

| **Barrier 21: There is delay in the routing (bottles with culture media arrive in the lab too late or not at all)** | | |
| --- | --- | --- |
| **Applicable to indicator: 1 - 3** | | |
| **Improvement strategy** | **Rationale or description** | **Supporting material** |
| 1. Implement a standard routing from ward to microbiology department | A standard routing, such as pneumatic tube transport might reduce delay in routing to the laboratory. |  |
| 13 + 14 + 15 + 23 |  |  |

| **Barrier 22: Patient safety hinders performance of blood culture performance before start of antibiotic therapy** | | |
| --- | --- | --- |
| **Applicable to indicator: 1** | | |
| **Improvement strategy** | **Rationale or description** | **Supporting material** |
| 13 + 14 + 15 |  |  |

| **Barrier 23: The PDMS / EHR hinders performance of the necessary actions for appropriate antibiotic use** | | |
| --- | --- | --- |
| **Applicable to indicator: 1 - 2 - 3** | | |
| **Improvement strategy** | **Rationale or description** | **Supporting material** |
| 1. Adapt the PDMS / EHR | Alert or reminder can increase appropriate antibiotic use, but be aware of alert fatigue [32]. |  |
| 19 + 20 + 21 |  |  |

1. **Capacity for organisational change**

| **Barrier 24: there is a lack of capable leadership** | | |
| --- | --- | --- |
| **Applicable to indicator: 1 - 2 - 3** | | |
| **Improvement strategy** | **Rationale or description** | **Supporting material** |
| 1. Provide external support or training for managers and leaders | It might be helpful to share knowledge with external contacts, because they might have another opinion or new perspective about certain topics. |  |
| 1. Shift or allocate leadership or management responsibilities to someone with a suitable style | Adequate leadership can lead to a more efficient and effective workflow. |  |
| 16 + 17 |  |  |

**REFERENCES**

[1] Schuts EC, Hulscher ME, Mouton JW, Verduin CM, Stuart JW, Overdiek HW, et al. Current evidence on hospital antimicrobial stewardship objectives: a systematic review and meta-analysis. The Lancet Infectious diseases 2016.

[2] Woolf SH, Grol R, Hutchinson A, Eccles M, Grimshaw J. Clinical guidelines: potential benefits, limitations, and harms of clinical guidelines. BMJ (Clinical research ed) 1999;318(7182):527-30.

[3] Kollef MH. Optimizing antibiotic therapy in the intensive care unit setting. Critical care (London, England) 2001;5(4):189-95.

[4] Lehman KD, Thiessen K. Sepsis guidelines: Clinical practice implications. Nurse Pract 2015;40(6):1-6.

[5] Barlam TF, Cosgrove SE, Abbo LM, MacDougall C, Schuetz AN, Septimus EJ, et al. Implementing an Antibiotic Stewardship Program: Guidelines by the Infectious Diseases Society of America and the Society for Healthcare Epidemiology of America. Clinical infectious diseases : an official publication of the Infectious Diseases Society of America 2016;62(10):e51-77.

[6] van Kasteren ME, Mannien J, Ott A, Kullberg BJ, de Boer AS, Gyssens IC. Antibiotic prophylaxis and the risk of surgical site infections following total hip arthroplasty: timely administration is the most important factor. Clinical infectious diseases : an official publication of the Infectious Diseases Society of America 2007;44(7):921-7.

[7] Cohen J, Brun-Buisson C, Torres A, Jorgensen J. Diagnosis of infection in sepsis: an evidence-based review. 2004.

[8] Cockerill FR, 3rd, Wilson JW, Vetter EA, Goodman KM, Torgerson CA, Harmsen WS, et al. Optimal testing parameters for blood cultures. Clinical infectious diseases : an official publication of the Infectious Diseases Society of America 2004;38(12):1724-30.

[9] Roberts JA, Norris R, Paterson DL, Martin JH. Therapeutic drug monitoring of antimicrobials. 2012.

[10] Udy AA, Roberts JA, Boots RJ, Paterson DL, Lipman J. Augmented renal clearance: implications for antibacterial dosing in the critically ill. Clin Pharmacokinet 2010;49(1):1-16.

[11] Oostdijk EAN. Selective decontamination in ICU patients: Dutch guideline. 2015.

[12] Vincent JL, Rello J, Marshall J, Silva E, Anzueto A, Martin CD, et al. International study of the prevalence and outcomes of infection in intensive care units. Jama 2009;302(21):2323-9.

[13] Centers for Disease Control and Prevention. Report: A public health action plan to combat antimicrobial resistance. Available from: <http://www.cdc.gov/drugresistance/pdf/public-health-action-plan-combat-antimicrobial-resistance.pdf>. 2011.

[14] Lewis CP, Corley DJ, Lake N, Brockopp D, Moe K. Overcoming Barriers to Effective Pain Management: The Use of Professionally Directed Small Group Discussions. Pain management nursing : official journal of the American Society of Pain Management Nurses 2014.

[15] Bero LA, Grilli R, Grimshaw JM, Harvey E, Oxman AD, Thomson MA. Closing the gap between research and practice: an overview of systematic reviews of interventions to promote the implementation of research findings. The Cochrane Effective Practice and Organization of Care Review Group. BMJ (Clinical research ed) 1998;317(7156):465-8.

[16] Jun J, Kovner CT, Stimpfel AW. Barriers and facilitators of nurses' use of clinical practice guidelines: An integrative review. Int J Nurs Stud 2016;60:54-68.

[17] Fischer F, Lange K, Klose K, Greiner W, Kraemer A. Barriers and Strategies in Guideline Implementation-A Scoping Review. Healthcare (Basel) 2016;4(3).

[18] Scales DC, Dainty K, Hales B, Pinto R, Fowler RA, Adhikari NK, et al. A multifaceted intervention for quality improvement in a network of intensive care units: a cluster randomized trial. Jama 2011;305(4):363-72.

[19] Erdek MA, Pronovost PJ. Improving assessment and treatment of pain in the critically ill. International journal for quality in health care : journal of the International Society for Quality in Health Care / ISQua 2004;16(1):59-64.

[20] Davey P, Brown E, Charani E, Fenelon L, Gould IM, Holmes A, et al. Interventions to improve antibiotic prescribing practices for hospital inpatients. The Cochrane database of systematic reviews 2013(4):Cd003543.

[21] Flodgren G, Parmelli E, Doumit G, Gattellari M, O'Brien MA, Grimshaw J, et al. Local opinion leaders: effects on professional practice and health care outcomes. The Cochrane database of systematic reviews 2011(8):CD000125.

[22] Grol R, Wensing M. What drives change? Barriers to and incentives for achieving evidence-based practice. Med J Aust 2004;180(6 Suppl):S57-60.

[23] Davey P, Marwick CA, Scott CL, Charani E, McNeil K, Brown E, et al. Interventions to improve antibiotic prescribing practices for hospital inpatients. The Cochrane database of systematic reviews 2017;2:CD003543.

[24] Evans RS, Classen DC, Pestotnik SL, Lundsgaarde HP, Burke JP. Improving empiric antibiotic selection using computer decision support. Archives of internal medicine 1994;154(8):878-84.

[25] Reed EE, Stevenson KB, West JE, Bauer KA, Goff DA. Impact of formulary restriction with prior authorization by an antimicrobial stewardship program. Virulence 2013;4(2):158-62.

[26] Conroy KM, Elliott D, Burrell AR. Testing the implementation of an electronic process-of-care checklist for use during morning medical rounds in a tertiary intensive care unit: a prospective before-after study. Ann Intensive Care 2015;5(1):60.

[27] van Daalen FV, Prins JM, Opmeer BC, Boermeester MA, Visser CE, van Hest RM, et al. Effect of an antibiotic checklist on length of hospital stay and appropriate antibiotic use in adult patients treated with intravenous antibiotics: a stepped wedge cluster randomized trial. Clinical microbiology and infection : the official publication of the European Society of Clinical Microbiology and Infectious Diseases 2017;23(7):485 e1- e8.

[28] Hatherley C, Jennings N, Cross R. Time to analgesia and pain score documentation best practice standards for the Emergency Department - A literature review. Australas Emerg Nurs J 2016;19(1):26-36.

[29] Pronovost P, Berenholtz S, Dorman T, Lipsett PA, Simmonds T, Haraden C. Improving communication in the ICU using daily goals. Journal of critical care 2003;18(2):71-5.

[30] Hulscher ME, Schouten LM, Grol RP, Buchan H. Determinants of success of quality improvement collaboratives: what does the literature show? BMJ quality & safety 2013;22(1):19-31.

[31] Patel SJ, Saiman L. Principles and strategies of antimicrobial stewardship in the neonatal intensive care unit. Semin Perinatol 2012;36(6):431-6.

[32] Thursky KA, Buising KL, Bak N, Macgregor L, Street AC, Macintyre CR, et al. Reduction of broad-spectrum antibiotic use with computerized decision support in an intensive care unit. International journal for quality in health care : journal of the International Society for Quality in Health Care / ISQua 2006;18(3):224-31.
